# *METTL3* and *FTO* Regulate Heat Stress Response in Hu Sheep Through Lipid Metabolism via m6A Modification

**DOI:** 10.3390/ani15020193

**Published:** 2025-01-13

**Authors:** Bowen Chen, Chao Yuan, Tingting Guo, Jianbin Liu, Zengkui Lu

**Affiliations:** 1 Key Laboratory of Animal Genetics and Breeding on Tibetan Plateau, Ministry of Agriculture and Rural Affairs, Lanzhou Institute of Husbandry and Pharmaceutical Sciences, Chinese Academy of Agricultural Sciences, Lanzhou 730050, China; chenbw202204@163.com (B.C.); yuanchao@caas.cn (C.Y.); guotingting@caas.cn (T.G.); 2 Sheep Breeding Engineering Technology Research Center of Chinese Academy of Agricultural Sciences, Lanzhou 730050, China

**Keywords:** m6A methylation, *METTL3*, *FTO*, heat stress, lipid metabolism, sheep

## Abstract

In an established hepatocyte lipid deposition heat stress model, the expression levels of *METTL3* and *FTO* were significantly upregulated (*p* < 0.05), indicating that *METTL3* and *FTO* play important roles in the process of lipid deposition heat stress in hepatocytes. Transcriptome and metabolome analyses showed that lipid deposition heat stress had significant effects on the linoleic acid, linolenic acid, glycerophospholipid, and arachidonic acid metabolic pathways in hepatocytes. After *METTL3* knockdown, the m6A methylation level decreased, but the difference was not significant (*p* > 0.05), the *FABP4* and *Accα* expression levels increased, and the *HSP60*, *HSP70*, and *HSP110* expression levels decreased significantly. After *METTL3* overexpression, the m6A methylation level increased significantly and the expression levels of *FABP4*, *ATGL*, *Accα*, *HSP60*, *HSP70*, *HSP90*, and *HSP110* decreased significantly, indicating that the overexpression of *METTL3* reduced the expression of heat shock genes by inhibiting the lipid-deposition-related gene expression in an m6A-dependent manner. The m6A methylation level increased significantly after *FTO* knockdown, while *HSP60*, *HSP110*, *FABP4*, *ATGL*, and Accα expression levels were significantly reduced. Following *FTO* overexpression, the m6A methylation level and *HSP60*, *HSP90*, and *HSP110* expression levels significantly decreased, while the *ATGL* and *Accα* expression levels significantly increased. This indicates that the overexpression of *FTO* promoted the expression of lipid-deposition-related genes in an m6A-dependent manner to reduce the expression of heat shock genes. Transcriptome and metabolome sequencing screened a large number of differential genes and metabolites, and a KEGG enrichment analysis showed that m6A methylation mainly regulated heat stress by affecting the TNF, cAMP, MAPK, lipolysis, and synthesis pathways in hepatocytes. In the lipid deposition heat stress model of preadipocytes, the regulation of gene expression was similar to that in hepatocytes.

## Simple Summary

Heat stress seriously affects the productivity, product quality, and health of animals and increases veterinary costs, feeding and management expenses, and animal welfare issues. Our previous study showed that m6A methylation is involved in regulating heat stress responses in Hu sheep and plays an important role in hepatic lipid metabolism; however, the exact underlying mechanisms remain unclear. Our study is the first to establish an in vitro lipid deposition heat stress model in primary hepatocytes and preadipocytes of Hu sheep. By knocking down or overexpressing the m6A methylation methylase (*METTL3*) and demethylase (*FTO*) genes in the lipid deposition heat stress models, we used RT-qPCR, BODIPY staining, RNA sequencing, and LC-MS techniques to reveal the molecular mechanisms by which m6A methylation modification mediates lipid metabolism to regulate heat stress in Hu sheep, providing a scientific basis and theoretical support for guiding the production of Hu sheep under heat stress. Our findings indicate that *METTL3* reduced lipid deposition in an m6A-dependent manner to alleviate heat stress, and *FTO* increased lipid deposition in an m6A-dependent manner to alleviate heat stress. m6A methylation mainly regulated Hu sheep heat stress through the TNF, cAMP, MAPK, lipolysis, and lipid synthesis pathways in hepatocytes.

## 1. Introduction

The United Nations Intergovernmental Panel on Climate Change issued a special report on global warming with noticeable and intensifying rising temperatures in 2022. This report emphasized that in the context of sustainable development, measures should be taken to respond to the threats posed by climate change [1]. Temperature is the most important ecological factor affecting livestock production. With global warming and the increasing frequency of extreme high-temperature weather events, heat stress (HS) will pose an increasingly prominent threat to animals [2].

Heat stress is a physiological and biochemical state experienced by animals in hot environments, especially when temperatures exceed their comfort range, and sheep exhibit a range of negative responses. Elevated body temperature caused by heat stress not only affects the milk yield of dairy cows but also reduces the milk yield and fat and protein content of dairy goats [3]. It affects oocyte maturation and reduces maternal conception and lambing rates during the mating period [4,5,6]. High temperatures lead to an increase in uterine temperature, which adversely affects fertilization and embryo survival, thereby affecting the pregnancy rate [4,7]. Secondly, heat stress increases drip loss and cooking loss in the muscle in high-temperature environments, which may be due to heat-stress-induced oxidative stress that disrupts cell membrane function and leads to increased permeability [8]. Heat stress also reduces the dry matter intake of dairy cows, sheep, pigs, and poultry, thereby negatively affecting nutrient uptake, which in turn affects the immune system and inflammatory response [9,10,11], causing an imbalance in the microbiome and an increase in diseases, which ultimately increases breeding costs. Previous studies have shown that heat stress increases lipid deposition in animals. High-temperature conditions can reduce lipid oxidation in pigs and promote the activity of lipoprotein lipase (LPL) in adipose tissue, thereby promoting lipid deposition [12,13]. Heat stress upregulates genes involved in fatty acid uptake and triglyceride synthesis and promotes triglyceride storage in adipocytes [14]. Heat stress also increases relative lipid deposition in broilers. Because heat stress promotes glucose production in the body, acetyl-coenzyme carboxylase is inhibited, leading to the production of more malonyl-CoA, thereby increasing lipid deposition in broilers [15]. During heat stress, basal levels of free fatty acids are often reduced in rodents, pigs, and dairy cows [16], which is thought to be a function of the animal’s maintenance of energy needs through reduced metabolic heat production, as well as increased lipolysis through reduced adaptive mechanisms resulting in lipid deposition to avoid additional thermal load [17,18]. This shows that when animals respond to heat stress, their lipid metabolism undergoes important changes [19,20,21].

The modification of m6A methylation plays a key role in the heat stress response in cows and sheep [22]. During heat stress in Hu sheep, m6A methylation resulted in changes in the overall characteristics of the methylation profile [23,24]. At the transcriptional level, the mRNA expression levels of *METTL3*, *METTL14*, *WTAP*, *FTO*, *ALKBH5*, *YTHDF1*, *YTHDF2*, *YTHDF3*, *YTHDC1*, and *YTHDC2* were significantly higher in Hu sheep in the heat stress group than those in the control group. The protein expression levels of sheep METTL14, WTAP, FTO, ALKBH5, YTHDF3, and YTHDC1 were significantly higher than those in the control group. Using MeRIP-Seq technology, researchers also found that the average number of m6A methylation modification sites in each transcript of Hu sheep in the heat stress group was significantly lower than that in the control group [23]. A functional enrichment analysis revealed that the key m6A methylation modification genes were significantly downregulated in heat stressed sheep. Genes in lipid-metabolism-related signaling pathways such as Wnt, TGF-β, and AMPK were enriched, revealing that m6A methylation modification is involved in regulating heat stress in Hu sheep through liver lipid metabolism, but the exact mechanism of action remains unclear. Therefore, it is particularly vital to study the mechanisms underlying heat stress responses in Hu sheep.

Previous studies have found that m6A methylation modification can regulate the heat stress response and lipid metabolism process of Hu sheep [25,26] and may regulate Hu sheep heat stress responses through liver lipid metabolism [23,24]. However, how m6A methylation modification regulates heat stress in Hu sheep through lipid metabolism has not yet been fully explored. This study firstly established a lipid deposition heat stress model in Hu sheep liver cells and adipocytes. By knocking down or overexpressing the m6A methylase *METTL3* and demethylase *FTO* genes in the model, and using RT-qPCR, BODIPY staining, and multi-omics technology to explored the molecular mechanism by which m6A methylation modification mediates lipid metabolism to regulate heat stress in Hu sheep. The research provided a scientific basis and theoretical support for guiding the production practices of Hu sheep under heat stress.

## 2. Materials and Methods

### 2.1. Cell Isolation, Culture, and Sample Collection

Three one-day-old newborn healthy Hu sheep (1.5–3 kg, ♂) from the Lanzhou Wanshan Plantation and Breeding Professional Cooperative (36°03′ N, 103°40′ E, average altitude 1530–1580 m) were used in this study. Those sheep were anesthetized with isoflurane inhalation (Sigma-Aldrich, St. Louis, MO, USA) and then fixed on a trough-shaped stool, and the carotid artery was cut off for bloodletting and slaughter. After the bloodletting was completed, a sterile sharp knife was used to pick up the sheepskin along the midline of the abdomen, and the livers and perirenal subcutaneous adipose tissue were obtained and immersed in 1×PBS (Solarbio, Beijing, China) containing 2% penicillin–streptomycin (PS, Gibco, Carlsbad, CA, USA) and then brought to the laboratory. The sheep primary hepatocyte and preadipocyte isolation and culture procedures were similar to those previously reported [25,27]. Briefly, sterile scissors were used to cut 1 × 1 mm^3^ liver tissues; then, 5 mL 0.25% trypsin (Gibco) and 0.1 mg/mL type IV collagenase (Sigma-Aldrich) in a ratio of 1:1 was added for digestion and incubated at 37 °C in a water bath for 15 min with continuous shaking until the digestion was complete. The cells were resuspended and cultured in 6-well plates at 37 °C in a 5% CO_2_ incubator after being filtered using a 100 μm sieve. Briefly, adipose tissue was cut into 1 × 1 mm^3^ tissue blocks and digested with 1 mg/mL type I collagenase (Sigma-Aldrich) at 37 °C for 60–90 min. The supernatant was centrifuged at 1500 rpm for 10 min after filtration with a 100 μm cell sieve. The cells were resuspended and passed through a 70 μm cell sieve, counted after being centrifuged, and cultured in a 5% CO_2_ incubator at 37 °C. Those cultures were tested and confirmed to be negative for mycoplasma contamination before use.

### 2.2. BODIPY Staining

The steps of BODIPY staining of cells were similar to that previously reported [26]. Briefly, cultured cells were washed with 1×PBS and fixed with 4% paraformaldehyde (Sigma-Aldrich) for 15 min at room temperature. After washing with 1×PBS, the cells were incubated with 1×PBS containing 1 μg/mL BODIPY 493/503 (CHEMEGEN, Shanghai, China) stain for 20 min and imaged using a ZEISS LSM800 confocal laser scanning microscope (Munich, Germany, Plan APOCHROMAT 10×/0.45). Image processing was carried out with ZEN 3.4 software.

### 2.3. Lipid Deposition Heat Stress (LD + HS) Assay

For lipid deposition, primary hepatocytes were incubated with a 1.2 mM fatty acid solution [oleic acid (OA, Sigma-Aldrich): palmitic acid (PA, Sigma-Aldrich) = 2:1] in William’s Medium E (Gibco) containing 15% FBS (Gibco) for 24 h (*n* = 3, three technical repetitions were performed for each sample). The primary preadipocytes were incubated with a cocktail of insulin (10 μg/mL, Sigma-Aldrich), dexamethasone (1 μM, Sigma-Aldrich), and 3-isobutyl-1-methylxanthine (0.5 mM, Sigma-Aldrich) in DMEM/F12 (Gibco) with 10% FBS for 2 d, followed by culture with DMEM/F12, 10% FBS, and insulin (10 μg/mL, Gibco) for another 2 d, and then cultured with DMEM/F12 and 10% FBS for 2 d (*n* = 3, three technical repetitions were performed for each sample) [26]. The medium was replaced with DMEM/F12 supplemented with 10% FBS for 2 d.

### 2.4. mRNA m6A Methylation Quantification

Relative mRNA m6A methylation was quantified using an EpiQuik mRNA m6A methylation quantification kit (Epigentek, St. Louis, MO, USA). Briefly, the principal operational steps were as follows: (1) buffer and solution preparation; (2) RNA binding; (3) m6A RNA capture; and (4) m6A calculation. The calculation of the percentage of m6A in total RNA was carried out using the formula m6A %$=\frac{(Sample\;OD-NC\;OD)÷S}{(PC\;OD-NC\;OD)÷P}$, where *S* is the amount of input sample RNA in ng, *P* is the amount of input positive control (PC) in ng, and NC is the native control (*n* = 3, three technical repetitions were performed for each sample).

### 2.5. Detection of Triglyceride (TG) Content

The content of TG was determined according to the instructions of the triglyceride (TG) kits (ZHONGSHENG, Beijing, China). Briefly, 4 μL of sample (or standard, *n* = 3) was added to 300 μL of R1 reagent and incubated at 37 °C for 5 min; then, 50 μL of R2 reagent was added and incubated at 37 °C for 5 min, and the reagent blank tube was used to correct zero. The absorbance A of the standard and sample at 500 nm was detected on a microplate reader. The calculation was carried out with the formula triglyceride concentration (mmol/L) = (A_sample concentration_/A_standard concentration_) × standard concentration, where the standard concentration is shown in the kit label.

### 2.6. Lentiviral Overexpression and RNAi Constructs and Infection of Cells

The target sequence for the short hairpin RNA (shRNA) interference of *METTL3* was 5′-GAGAGCCTTCTTAACCAACAA-3′, and that for the interference of *FTO* was 5′-ACGGTGAAATCTCTTTGAAAT-3′. The primer sequence information on the *METTL3* and *FTO* overexpression constructs refer to [25]. The synthesis and packaging of lentiviruses and the native control (NC) were performed by Genepharma Biotech Co. (Shanghai, China).

For the overexpression and interference experiments, the primary hepatocytes were washed with PBS and then transfected with *METTL3* overexpression (M3-OE), *FTO* overexpression (FTO-OE), *METTL3* shRNA (M3-sR), *FTO* shRNA (FTO-sR), or NC lentivirus constructs for 72 h. Cells were isolated, and transfection efficiency was confirmed by RT-qPCR (*n* = 3, three technical repetitions were performed for each sample).

### 2.7. RNA Sequencing (RNA-Seq) Data and Analysis

The methods and steps of the RNA-seq data analysis were similar to that previously reported [26]. Briefly, the extracted total RNA was checked using RNase-free agarose gel electrophoresis, and the libraries were constructed using a VAHTS Universal V6 RNA-seq Library Prep Kit by OE Biotech Co., Ltd. (Shanghai, China). The libraries were sequenced on an Illumina Novaseq6000 platform (Illumina, San Diego, CA, USA), and 150 bp paired-end reads were generated. Raw reads in fastq format were firstly processed using fastp [28]. The clean reads were mapped to the reference genome using HISAT2 [29]. The FPKM [30] of each gene was calculated, and the read counts of each gene were obtained by HTSeq-count [31]. Differential expression genes (DEGs, *p* < 0.05 and |log-fold change| ≥ 1) between two groups were analyzed using DESeq2 [32]. The KEGG pathway enrichment analyses of the DEGs were performed using R based on hypergeometric distribution. Pathways with a value of *p* < 0.05 were considered significantly enriched.

### 2.8. Ultra-High-Performance Liquid Chromatography–Mass Spectrometry (LC-MS) Analysis

The methods and steps of the RNA-seq data analysis were similar to those previously reported [26]. Briefly, the lipid was first extracted by 600 μL chloroform–methanol (2:1, *v*/*v*), and then about 200 μL of chloroform layers was transferred into a centrifuge tube after centrifugation for 10 min at 13,000 rpm and 4 °C. Finally, the samples were reconstituted in isopropanol–methanol (1:1, *v*/*v*). The metabolomic data analysis was performed by Shanghai Luming biological technology co., LTD (Shanghai, China). The LC system was performed using an ExionLC™ System. The temperature of the autosampler and oven were set at 4 °C and 55 °C, respectively. The sample injection volume was 5 μL. The flow rate of eluents was set at 0.35 mL/min. The positive and negative data were combined to obtained combined data, which were imported into the R ropls package. Differential metabolites were further used for the KEGG pathway (http://www.genome.jp/kegg/, accessed on 19 November 2023) enrichment analysis.

### 2.9. Joint Analysis of Transcriptomic and Metabolomic Data

Pearson correlation coefficients were calculated between the differential metabolites and DEGs via pairwise comparison using the Hmisc package in R^1^ (https://cran.r-project.org/web/packages/Hmisc/index.html, accessed on 23 November 2023). DEGs and differential metabolites with a threshold of |r| > 0.9 and *p* < 0.05 were considered significantly correlated and were subjected to conjoint biological annotation using the KEGG database. The results were visualized with the OmicShare tool, an online platform for data analysis (https://www.omicshare.com/tools/Home/Soft/enrich_circle, accessed on 28 February 2024).

### 2.10. Reverse Transcription, RT-qPCR, and Statistical Analysis

A TransGen Biotech reverse transcription kit (Transgen, Beijing, China, refer to the instructions for specific methods) was used to reverse-transcribe extracted RNA into cDNA at 42 °C for 15 min and 85 °C for 5 s. The cDNA was stored at −20 °C. RT-qPCR was performed in 20 μL volumes per the manufacturer’s protocol (TransStar Tip Green qPCR SuperMix, Transgen, Beijing, China) on a Bio-Rad C1000 Thermal Cycler using the following conditions: 94 °C for 30 s and 40 cycles of 94 °C for 5 s, 60 °C for 15 s, and 72 °C for 10 s. *β-actin* was used as a reference gene to normalize gene expression. Supplementary Table S1 lists the primer sequences used for RT-qPCR. The Shapiro–Wilk test was used to check the normal distribution. The Brown–Forsythe test was used to check the homogeneity of variance. Statistical analyses were performed using Student’s *t*-test between two groups and using a one-way analysis of variance among three groups by SPSS 22 software (http://www.spss.com, accessed on 13 December 2024). The results were displayed as mean ± SD by GraphPad Prism 8 software (https://www.graphpad.com/, accessed on 13 December 2024). A value of *p* < 0.05 was considered significant. Groups marked with the same letter were considered to have no significant difference, and those without the same letter were significantly different (*p* < 0.05).

## 3. Results

### 3.1. Establishment of an in Vitro Lipid Deposition and Heat Stress Model in Primary Hepatocytes and Preadipocytes

**Figure 1:**
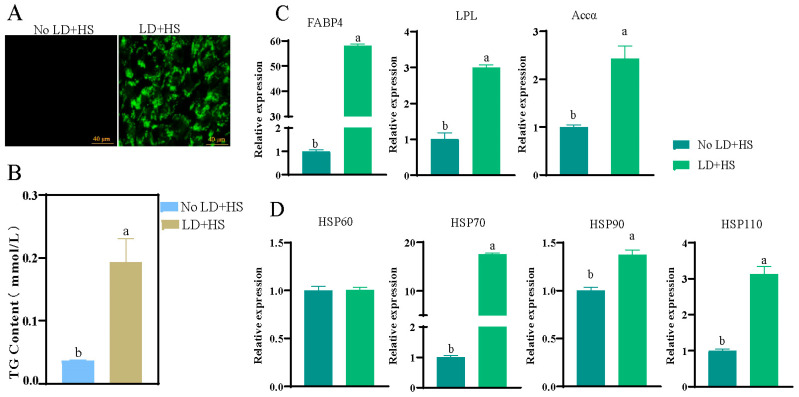
Detection of the changes in lipid deposition and heat shock gene expression with or without LDHS in primary hepatocytes: (**A**) detection of lipid droplet formation by BODIPY staining, scale bar = 20 μm; (**B**) detection of TG content; (**C**) expression of genes related to lipid metabolism; (**D**) expression of genes related to heat stress. Groups marked with the same letter were considered to have no significant difference, and those without the same letter were significantly different (*p* < 0.05).

A lipid deposition heat stress model was established by treating primary hepatocytes with heat stress for 1 h after 24 h of lipid deposition. The BODIPY staining results showed that compared with the control group (No LD + HS: normally cultured primary hepatocytes with no lipid deposition or heat stress treatment), the green fluorescence of hepatocytes was significantly enhanced after lipid deposition and heat stress (LD + HS; Figure 1A). The TG content and the expression levels of lipid-metabolism-related genes (*FABP4*, *LPL*, and *Accα*) and heat-shock-related genes (*HSP70*, *HSP110*, and *HSP110*) were all significantly increased (*p* < 0.05, Figure 1B–D). These results indicate that the LD + HS model was successfully established in primary hepatocytes. Similarly, the preadipocytes were subjected to lipid deposition for 6 days and then subjected to heat stress treatment for 2 days to establish an LD + HS model. The BODIPY staining results showed that compared with the control group (No LD + HS: normally cultured preadipocytes with no LD + HS treatment), the green fluorescence after LD + HS was significantly enhanced (Figure S1A), the TG content was significantly increased (*p* < 0.05, Figure S1B), the expression levels of the lipid-metabolism-related genes *FABP4* and *LPL* were significantly increased (*p* < 0.05, Figure S1C), and the expression levels of the heat-stress-related genes *HSP60*, *HSP70*, *HSP90*, and *HSP110* were significantly increased (*p* < 0.05, Figure S1D). These results indicate that an LD + HS model was successfully established in the preadipocytes.

### 3.2. Effects of LD + HS on Gene Expression and Metabolites

**Figure 2:**
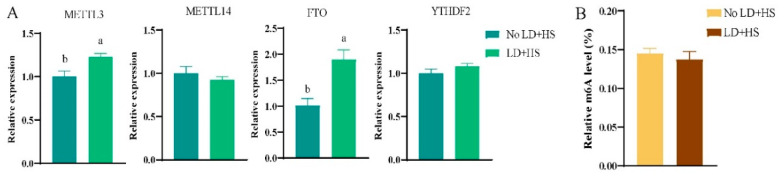
Effect of ADHS on m6A methylation level and gene expression in primary hepatocytes: (**A**) detection of m6A-methylation-related gene expression; (**B**) detection of m6A methylation level. Groups marked with the same letter were considered to have no significant difference, and those without the same letter were significantly different (*p* < 0.05).

Compared to the control group, the expression levels of the m6A-methylation-modification-related genes *METTL3* and *FTO* were significantly increased after LD + HS treatment in primary hepatocytes; however, there was no significant difference in the expression of *METTL14* and *YTHDF2* (*p* > 0.05, Figure 2A). These results indicate that *METTL3* and *FTO* play important roles in lipid deposition and heat stress in hepatocytes. The expression levels of *METTL3*, *METTL14*, *FTO*, and *YTHDF2* in preadipocytes were significantly increased after LD + HS treatment (*p* < 0.05, Figure S2A). The methylation level of m6A was significantly decreased (*p* < 0.05, Figure S2B), similar to the results obtained in primary hepatocytes.

**Figure 3:**
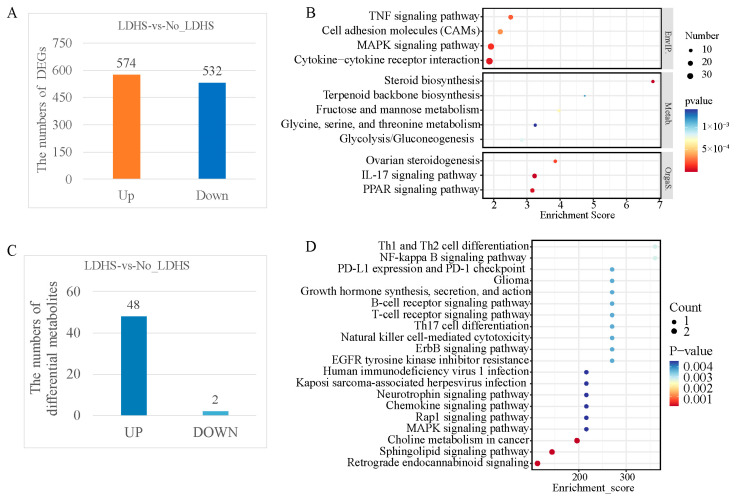
Transcriptomic and metabolic profiles with or without LDHS in primary hepatocytes: (**A**) histogram of the number of DEGs in the LDHS vs. no-LDHS group; (**B**) bubble diagram of the KEGG pathway enrichments for the DEGs in the LDHS vs. no-LDHS group; (**C**) histogram of the number of differential metabolites in the LDHS vs. no-LDHS group; (**D**) bubble diagram of the top 20 KEGG pathway enrichments for differential metabolites in the LDHS vs. no-LDHS group.

To further investigate the changes in gene and metabolite expression and cell signaling pathways after the LD + HS treatment in hepatocytes, we performed transcriptomic and metabolomic sequencing of primary hepatocytes with or without the LD + HS treatment. The quality control information of the transcriptome sequencing data is provided in Supplementary Table S2. The results show that the sequencing quality of the sample libraries was good and could be used for subsequent bioinformatic statistical analyses. For each sample, more than 96% of the reads were mapped onto the reference genome. We identified 1106 DEGs with or without LD + HS, comprising 574 upregulated and 532 downregulated genes (Figure 3A). These DEGs were mainly enriched in immune- and fatty-acid-metabolism-related pathways, such as the IL-17, TNF, and PPAR pathways (Figure 3B). Compared to the metabolites in the non-LD + HS group, we detected 50 differential metabolites, including 48 upregulated and 2 downregulated metabolites (37 positive and 13 negative ions, Figure 3C). The KEGG enrichment analysis showed that LD + HS had a significant effect on the lipid-metabolism-related pathways in the primary hepatocytes, including the linoleic acid and linolenic acid metabolic pathways and the sphingolipid signaling pathway (Figure 3D).

The Sankey diagram shows the interactions between differentially expressed genes and metabolites. Among them, genes such as heat shock protein family A member 8 (*HSPA8*), interleukin 6 (*IL6*), and solute carrier family 16 member 3 (*SLC16A3*) were closely related to the levels of metabolites such as TAG56:6 (16:0) and TAG56:7 (16:0). The KEGG enrichment analysis of the differentially expressed genes and metabolites showed that they had significant effects on lipid-metabolism-related pathways (*p* < 0.05), including the lipid decomposition, arachidonic acid metabolism, sphingolipid metabolism, and mitogen-activated protein kinase (MAPK) signaling pathways (Figure S3B).

### 3.3. The Molecular Mechanism of METTL3 Regulating Heat Stress Through Lipid Metabolism in Primary Hepatocytes and Preadipocytes

**Figure 4:**
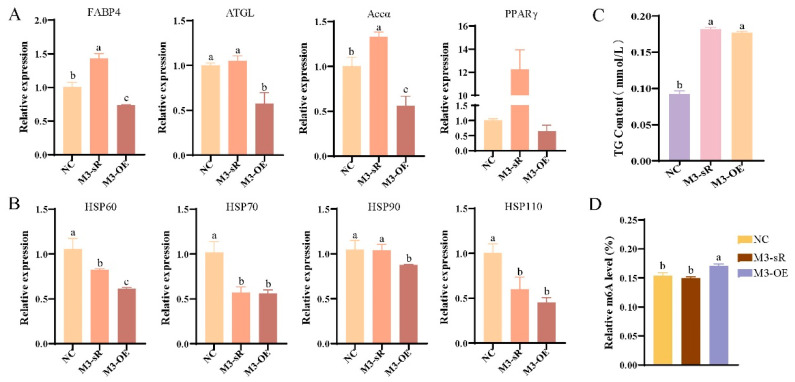
Effects of *METTL3* interference and overexpression on LDHS in primary hepatocytes: (**A**) detection of lipid-metabolism-related gene expression levels; (**B**) detection of heat-stress-related gene expression levels; (**C**) detection of m6A methylation level; (**D**) detection of TG content. Groups marked with the same letter were considered to have no significant difference, and those without the same letter were significantly different (*p* < 0.05).

The RT-qPCR analysis showed that compared to the NC, *FABP4* and *Accα* gene expression was significantly upregulated after *METTL3* knockdown, whereas *FABP4*, *ATGL*, and *Accα* gene expression was significantly reduced after *METTL3* overexpression (*p* < 0.05, Figure 4A). *HSP60*, *HSP70*, and *HSP110* expression significantly decreased after *METTL3* knockdown, whereas *HSP60*, *HSP70*, *HSP90*, and *HSP110* expression significantly increased after *METTL3* overexpression (*p* < 0.05, Figure 4B). The TG content significantly increased after *METTL3* knockdown or overexpression (*p* < 0.05, Figure 4C). The m6A methylation level decreased after knockdown of *METTL3*, but the difference was not significant (*p* > 0.05), whereas the m6A methylation level increased significantly after the overexpression of *METTL3* (*p* < 0.05, Figure 4D). These results indicate that overexpression of the *METTL3* gene inhibits the expression of lipid-metabolism-related genes in an m6A-dependent manner to reduce the expression of heat shock genes. The expression levels of *FABP4*, *Accα*, and *PPARγ* were significantly increased after the knockdown of *METTL3* in preadipocytes (*p* < 0.05), whereas *FABP4*, *ATGL*, *Accα*, and *LPL* were significantly decreased after the overexpression of *METTL3* (*p* < 0.05, Figure S4A). The expression levels of *HSP60*, *HSP70*, and *HSP110* were significantly decreased after *METTL3* knockdown or overexpression (*p* < 0.05, Figure S4B). Additionally, after *METTL3* knockdown, the TG content was significantly increased (*p* < 0.05, Figure S4C), and the m6A methylation level was significantly decreased (*p* < 0.05, Figure S4D), similar to the results observed in primary hepatocytes.

**Figure 5:**
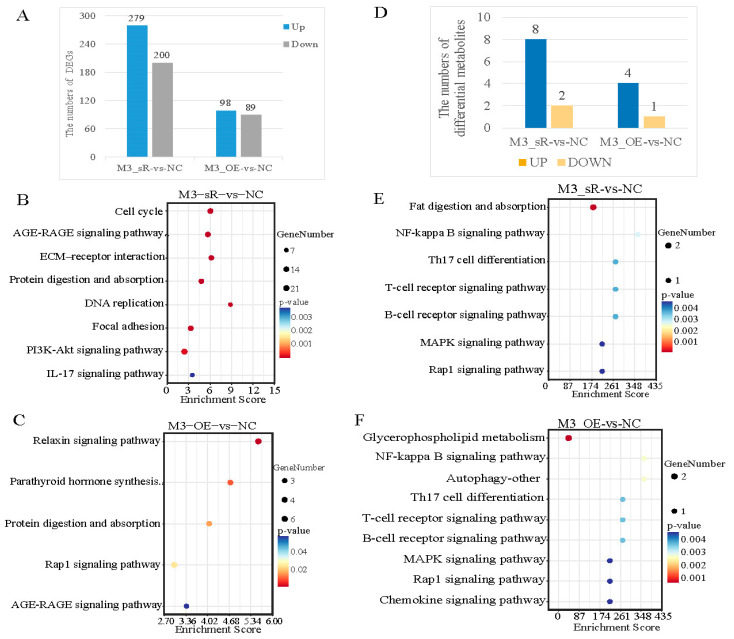
Transcriptome and metabolomics analysis of *METTL3* interference and overexpression on LDHS in primary hepatocyte: (**A**) histogram of the number of differentially expressed genes for the M3_sR vs. NC group and M3_OE vs. NC group; (**B**) bubble diagram of the KEGG enrichment analysis of DEGs in the M3_sR vs. NC group; (**C**) bubble diagram of the KEGG enrichment analysis of DEGs in the M3_OE vs. NC group; (**D**) histogram of differential metabolite quantities for the M3_sR vs. NC group and M3_OE vs. NC group; (**E**) bubble diagram of the top 20 KEGG enrichment analysis of differential metabolites in the M3_sR vs. NC group; (**F**) bubble diagram of the top 20 KEGG enrichment analysis of differential metabolites in the M3_OE vs. NC group.

Transcriptome sequencing was performed after the knockdown and overexpression of *METTL3* in the primary hepatocyte LD + HS model. Compared to the NC group, 279 differentially upregulated and 200 differentially downregulated genes were identified after *METTL3* knockdown, and 98 differentially upregulated and 89 differentially downregulated genes were identified after *METTL3* overexpression (Figure 5A). In the *METTL3* overexpression group, the KEGG enrichment analysis of differentially expressed genes showed that, compared to the NC group, these genes were mainly enriched in the PI3K-Akt, IL-17, and cell cycle pathways (Figure 5B). Differentially regulated genes were enriched in the linoleic acid metabolism, steroid hormone biosynthesis, and retinol metabolism pathways after *METTL3* knockdown (Figure 5C). The primary hepatocyte LD + HS model with *METTL3* knockdown and overexpression was subjected to LC-MS/MS-targeted metabolome analyses. Compared with the NC group, eight upregulated and two downregulated metabolites were screened after *METTL3* knockdown (eight in the positive mode and two in the negative mode). In the *METTL3* overexpression group, four upregulated and one downregulated metabolite were screened (one in positive mode and four in negative mode, Figure 5D). The KEGG enrichment analysis showed that the differential metabolites were enriched in the MAPK, Rap1, and Nf-kB signaling pathways after the knockdown or overexpression of *METTL3* (Figure 5E,F).

**Figure 6:**
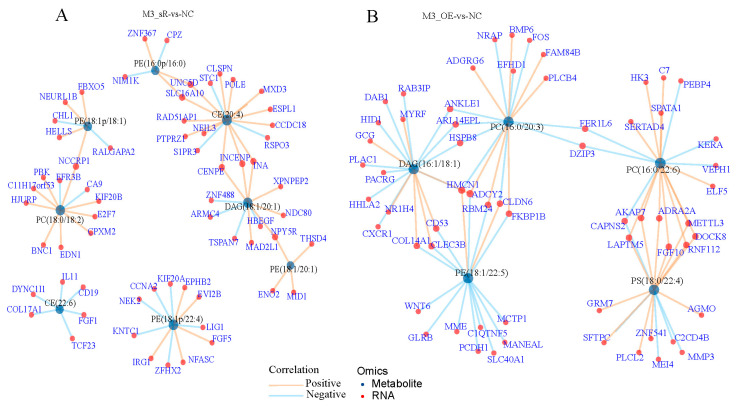
Relationship network diagram of differentially expressed genes and metabolites after *METTL3* interference and overexpression in primary hepatocytes: (**A**) network chart of differentially expressed genes and metabolites in the M3_sR vs. NC group; (**B**) network chart of differentially expressed genes and metabolites in the M3_OE vs. NC group.

Correlations between the differentially expressed genes and their metabolites were analyzed. In the *METTL3* knockdown (M3_sR) vs. NC group, genes such as solute carrier family 16 member 10 (*SLC16A10*) and protein tyrosine phosphatase receptor type Z1 (*PTPRZ1*) jointly regulated CE, DAG, and PE metabolites, and most genes positively regulated these metabolites. In addition, CE, DAG, and PE were linked by carbonic anhydrase 11 (*CA11*), inner centromere protein (*INCENP*), and neuropeptide Y receptor Y5 (*NPY5R*) genes to form a mutual regulatory relationship (Figure 6A). In the *METTL3* overexpression (M3_OE) vs. NC group, genes such as solute carrier family 40 member 1 (*SLC40A1*), protocadherin 1 (*PCDH1*), FKBP prolyl isomerase 1 B (*FKBP1B*), heat shock protein family B member 8, (*HSPB8*), Fer-1 like family member 6 (FER1L6), and *METTL3* formed complex correlation networks with the differential metabolites PE, DAG, PC, and PS. Among them, *METTL3* directly regulated PC and PS and indirectly regulated metabolites such as PE and DAG through other genes (Figure 6B).

### 3.4. The Molecular Mechanism of FTO Regulating Heat Stress Through Lipid Metabolism in Primary Hepatocytes and Preadipocytes

**Figure 7:**
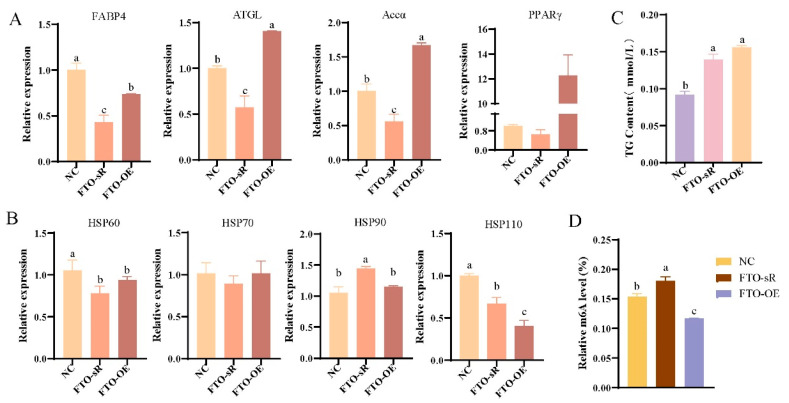
Effect of *FTO* interference and overexpression on lipid deposition in primary hepatocytes: (**A**) detection of the lipid-metabolism-related gene expression level; (**B**) detection of mRNA m6A methylation level; (**C**) detection of triglyceride content; (**D**) detection of m6A methylation level. Groups marked with the same letter were considered to have no significant difference, and those without the same letter were significantly different (*p* < 0.05).

The RT-qPCR analysis showed that, compared with the NC, *FABP4*, *ATGL*, and *Accα* gene expression was significantly downregulated after *FTO* knockdown, whereas the expression of *ATGL* and *Accα* genes was significantly increased after the overexpression of *FTO* (Figure 7A). The expression of *HSP60* and *HSP110* significantly decreased after *FTO* knockdown (*p* < 0.05), and the expression of *HSP90* significantly increased (*p* < 0.05), whereas in the *FTO* overexpression group, the expression levels of *HSP60*, *HSP90*, and *HSP110* significantly decreased (*p* < 0.05, Figure 7B). TG contents significantly increased after *FTO* knockdown or overexpression (*p* < 0.05, Figure 7C). In addition, m6A methylation levels also significantly increased after the *FTO* knockdown and significantly decreased after *FTO* overexpression (Figure 7D), indicating that *FTO* overexpression promotes the expression of lipid-metabolism-related genes in an m6A-dependent manner to reduce heat-shock-related gene expression. The expression levels of *FABP4* and *ATGL* were significantly decreased after *FTO* knockdown in preadipocytes (*p* < 0.05), whereas the expression levels of *FABP4*, *Accα*, *PPARγ*, and *LPL* were significantly increased after the overexpression of *FTO* (*p* < 0.05, Figure S5A). The expression of *HSP60* and *HSP110* decreased significantly after the overexpression of *FTO* (*p* < 0.05, Figure S5B), the TG content significantly increased (*p* < 0.05), and the m6A methylation level significantly decreased (*p* < 0.05, Figure S5C,D), similar to the results observed in primary hepatocytes.

**Figure 8:**
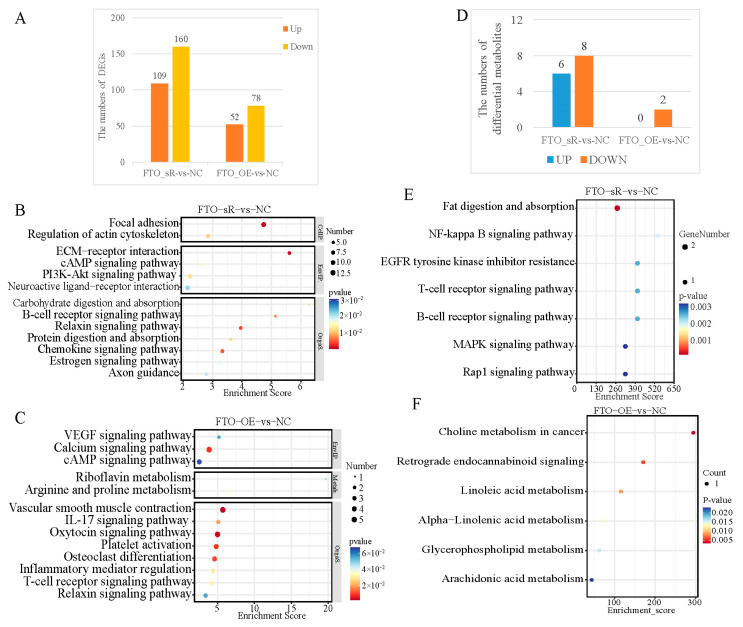
Transcriptomic and metabolic profiles of *FTO* interference and overexpression in primary hepatocytes: (**A**) histogram of the number of differentially expressed genes for the FTO_sR vs. NC group and FTO_OE vs. NC group; (**B**) bubble diagram of the KEGG pathway enrichment analysis in the FTO_sR vs. NC group; (**C**) bubble diagram of the KEGG pathway enrichment analysis in the FTO_OE vs. NC group; (**D**) histogram of the number of differential metabolites for the FTO_sR vs. NC group and FTO_OE vs. NC group; (**E**) bubble diagram of the top 20 KEGG enrichment analysis of differential metabolites in the FTO_sR vs. NC group; (**F**) bubble diagram of the KEGG enrichment analysis of differential metabolites in the FTO_OE vs. NC group.

The RNA-seq analysis of primary hepatocytes in the LD + HS model after the knockdown or overexpression of *FTO* identified 109 upregulated and 160 downregulated genes after *FTO* knockdown and 52 upregulated and 78 downregulated genes after *FTO* overexpression, compared with those in the NC group (Figure 8A). The KEGG enrichment analysis of differentially expressed genes showed that compared to the NC group, these genes were enriched in the cAMP, PIK3-Akt, and B-cell receptor pathways (Figure 8B). Differentially regulated genes were enriched in the VEGF, cAMP, and IL-17 pathways in the *FTO* overexpression (FTO_OE) vs. NC group (Figure 8C). An LC-MS metabolomic analysis was performed on primary hepatocytes from the LD + HS model with knockdown or overexpression of *FTO*. Compared to the NC group, 6 upregulated and 8 downregulated metabolites were screened after *FTO* knockdown (12 in positive mode and 2 in negative mode), and 2 downregulated metabolites were screened after the overexpression of *FTO* (1 in positive mode and 1 in negative mode; Figure 8D). The KEGG enrichment analysis showed that the differentially expressed metabolites were mainly enriched in pathways such as the MAPK signaling, lipid metabolism and uptake, and oleic acid metabolism pathways (Figure 8E,F).

**Figure 9:**
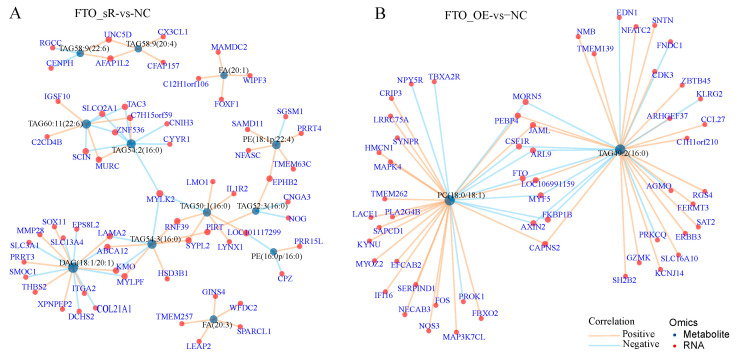
Relationship network diagram of differentially expressed genes and metabolites on *FTO* interference and overexpression in primary hepatocytes: (**A**) network chart of differentially expressed genes and metabolites in the FTO_sR vs. NC group; (**B**) network chart of differentially expressed genes and metabolites in the FTO_OE vs. NC group.

Joint transcriptomic and metabolomic analyses showed that immunoglobulin superfamily member 10 (*IGSF10*), zinc finger protein 536 (*ZNF536*), myosin light chain kinase 2 (*MYLK2*), and protogenin (*PRTG*) formed a mutual regulatory network relationship with metabolites such as TAG60:11 and TAG54:2 after *FTO* knockdown (Figure 9A). Upon the overexpression of *FTO*, the relationship between PC and TAG49:2 was regulated by MORN repeat containing 5 (*MORN5*), phosphatidylethanolamine binding protein 4 (*PEBP4*), junction adhesion molecule-like (*JAML*), and *FTO*, and most of the genes were positively correlated with metabolites (Figure 9B).

## 4. Discussion

Heat stress can cause the rates of feed intake and feed utilization in livestock animals to decline, which eventually leads to decreased animal production efficiency and immune function and increased mortality, resulting in huge economic losses and problems in maintaining animal welfare [2,33,34,35]. Consequently, research is increasingly focused on exploring the molecular mechanisms of heat stress regulation in animals to reduce its impact on livestock production through nutritional or gene regulation [36], which is of great importance for the healthy development of the animal husbandry industry. The expression levels of methylation-related genes also change significantly after lipid deposition heat stress, indicating that the m6A methylation gene plays an important regulatory role in the process of lipid deposition heat stress; however, the specific gene regulatory mechanism remains to be further studied.

Hu sheep and other animals undergo a series of reactions after heat stress, including increased respiratory rate, increased body temperature, and enhanced metabolism [37]. Previous studies have shown that heat stress reduces the lipid decomposition rate and increases lipid deposition in broilers [38,39]. Similarly, heat stress can reduce lipid oxidation and promote lipid deposition in piglets [12,13,14,15,16], indicating that animals can regulate heat stress through lipid metabolism. Our previous studies have also found that m6A methylation modification may regulate the heat stress response of Hu sheep through lipid metabolism. Studies have shown that the *METTL3* and *FTO* genes play important roles in regulating animal heat stress and lipid metabolism [23]. To analyze the specific regulatory mechanisms of these genes, we detected the mRNA methylation levels, TG content, and lipid-metabolism- and heat-shock-related gene expression levels by knocking down and overexpressing *METTL3* and *FTO* in an LD + HS cell model. The results showed that the m6A methylation level significantly increased after the overexpression of *METTL3*, whereas the expression of lipid-deposition-related genes significantly decreased. In addition, the expression of heat-shock-related genes was significantly decreased, showing similar results and consistent regulatory trends in preadipocytes, indicating that the overexpression of *METTL3* may alleviate heat stress by reducing lipid deposition at the transcriptome level in an m6A-dependent manner. However, after *FTO* overexpression, the m6A methylation level significantly decreased, the lipid-deposition-related gene expression level significantly increased, and the heat-shock-related gene expression level significantly decreased, indicating that the overexpression of *FTO* may increase lipid deposition at the transcriptome level by relying on m6A methylation to alleviate heat stress. However, the position of the m6A methylation modification and expression abundance of heat-shock-related and lipid-metabolism-related genes in this regulatory process remain to be explored. There was no statistically significant difference in m6A methylation levels after *METTL3* knockdown, which may indicate a compensatory effect of other m6A methyltransferases, which requires further study.

During heat stress, the basic levels of free fatty acids in cows are usually reduced [40], which is considered to be an adaptive mechanism for animals to maintain energy demands by reducing metabolic heat production and to avoid additional heat load by reducing lipolysis and increasing lipid deposition [17,18]. The m6A demethylase *FTO*, as an important gene related to regulating obesity, plays an important role in regulating animal fat metabolism. The level of m6A methylation modification in mRNA is highly negatively correlated with the animal obesity phenotype. Studies have shown that the overexpression of *METTL3* can increase m6A methylation levels and inhibit lipid deposition, while the overexpression of *FTO* can reduce m6A methylation levels and promote lipid deposition [14,41]. Our findings are consistent with Wang et al.’s study. However, whether the overexpression of *METTL3* or *FTO* genes will affect the methylation modification sites and abundance of heat-shock-related genes and lipid-metabolism-related genes, as well as the stability of modified mRNA, these detailed internal regulatory mechanisms still require the use of MeRIP-seq technology for further research. In addition, betaine, also known as trimethylglycine, is an important methyl donor that regulates DNA and m6A methylation modification. Studies have also shown that betaine can promote lipid metabolism in laying hens, reduce the abdominal lipid rate, and significantly reduce the lipid content in the liver [42,43]. It is expected to add m6A methyl donors (such as betaine) and methyl inhibitors (such as cycloleucine) in cells [44] or animal models to further study the regulatory mechanism of m6A methylation modification on heat stress molecules in Hu sheep. It can be used as a feed additive to develop anti-heat-stress feed, thereby providing a scientific basis and theoretical support for the feeding and management of Hu sheep under heat stress.

## 5. Conclusions

In conclusion, this study first established an in vitro lipid deposition heat stress model of Hu sheep primary hepatocytes and preadipocytes and then deeply analyzed the molecular mechanism of m6A-methylation-modification-mediated lipid metabolism to regulate heat stress in Hu sheep from the perspective of epigenetics. It was found that the *METTL3* and *FTO* genes regulate the heat stress of Hu sheep in an m6A-dependent manner and the TNF, cAMP, MAPK, lipolysis, and synthesis pathways. In the next step of this research, MeRIP-seq technology will be performed to further study whether m6A methylation modification regulates the response of Hu sheep hepatocytes to heat stress by affecting the methylation modification sites of heat-shock-related genes and lipid-metabolism-related genes.

## Data Availability

The data presented in this study are deposited in the SRA repository (https://www.ncbi.nlm.nih.gov/sra/?term=, accessed on 24 September 2024), accession number PRJNA1178813, and our data have been released.

## Supplementary Materials

The following supporting information can be downloaded at https://www.mdpi.com/article/10.3390/ani15020193/s1. Figure S1: Detection of lipid deposition and heat shock gene expression before and after ADHS in preadipocytes; Figure S2: Effect of ADHS on m6A methylation in preadipocytes; Figure S3: Joint analysis of transcriptome and metabolome; Figure S4: Effects of interference and overexpression of *METTL3* on ADHS of preadipocytes; Figure S5: Effects of interference and overexpression of *FTO* on ADHS in preadipocytes. Table S1: Primer sequence information; Table S2: Summary of sequencing RNA-seq data.

## Abbreviations

Accα: acetyl-coenzyme A carboxylase α; ATGL: adipose triglyceride lipase; DEGs: differentially expressed genes; FABP4: fatty acid binding protein 4; FTO: fat mass and obesity associated protein; GO: Gene Ontology; KEGG: Kyoto Encyclopedia of Genes and Genomes; LDHS: lipid deposition heat stress; m6A: N6-methyladenosine; METTL3: methyltransferase-like protein 3; METTL14: methyltransferase-like protein 14; PPARγ: peroxisome proliferator-activated receptor γ; RT-qPCR: quantitative reverse transcriptase-PCR; YTHDF2: YTH domain family proteins 2.
