# Supplementary material for: METTL3 and FTO Regulate Heat Stress Response in Hu Sheep Through Lipid Metabolism via m6A Modification
Source: Animals (Basel). 2025 Jan 13;15(2):193. doi: 10.3390/ani15020193 (PMC11758659; doi:10.3390/ani15020193)
Supplement: Supplementary file 1 [file animals-15-00193-s001.zip › Supplemental figures-revised.pdf]

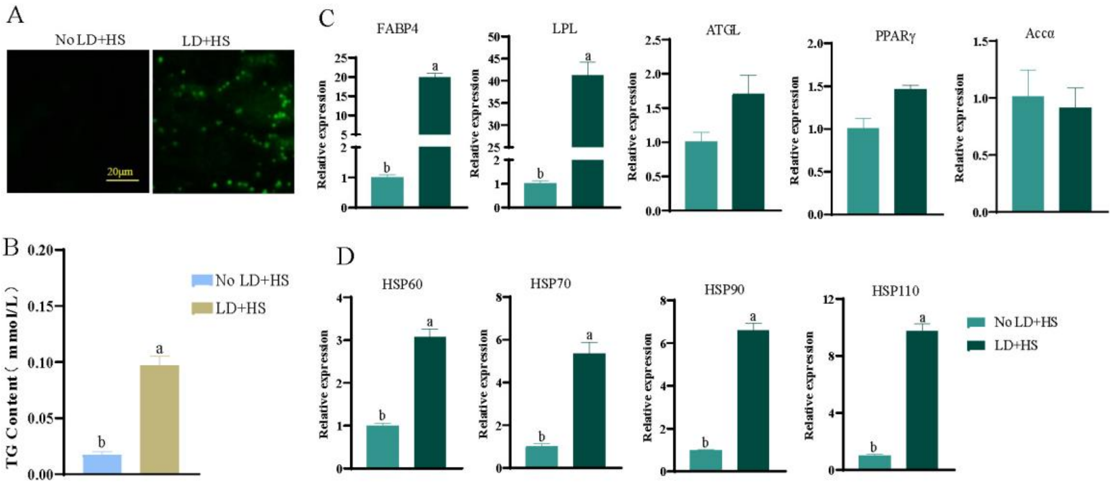

Figure S1 Detection of lipid deposition and heat shock gene expression before and after ADHS in preadipocytes. (A) Detection of fat deposition by bodipy staining, scale bar=20 μm; (B) Detection of intracellular triglyceride content; (C) Determination of mRNA m6A methylation level; (D) Expression of genes related to lipid deposition; (E) Expression of genes related to heat stress.

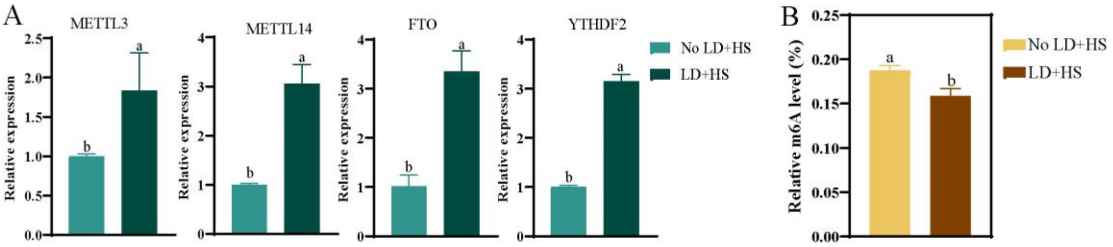

Figure S2 Effect of ADHS on m6A methylation in in preadipocytes. (A) Detection of m6A methylation-related gene expression; (B) Detection of m6A methylation level.

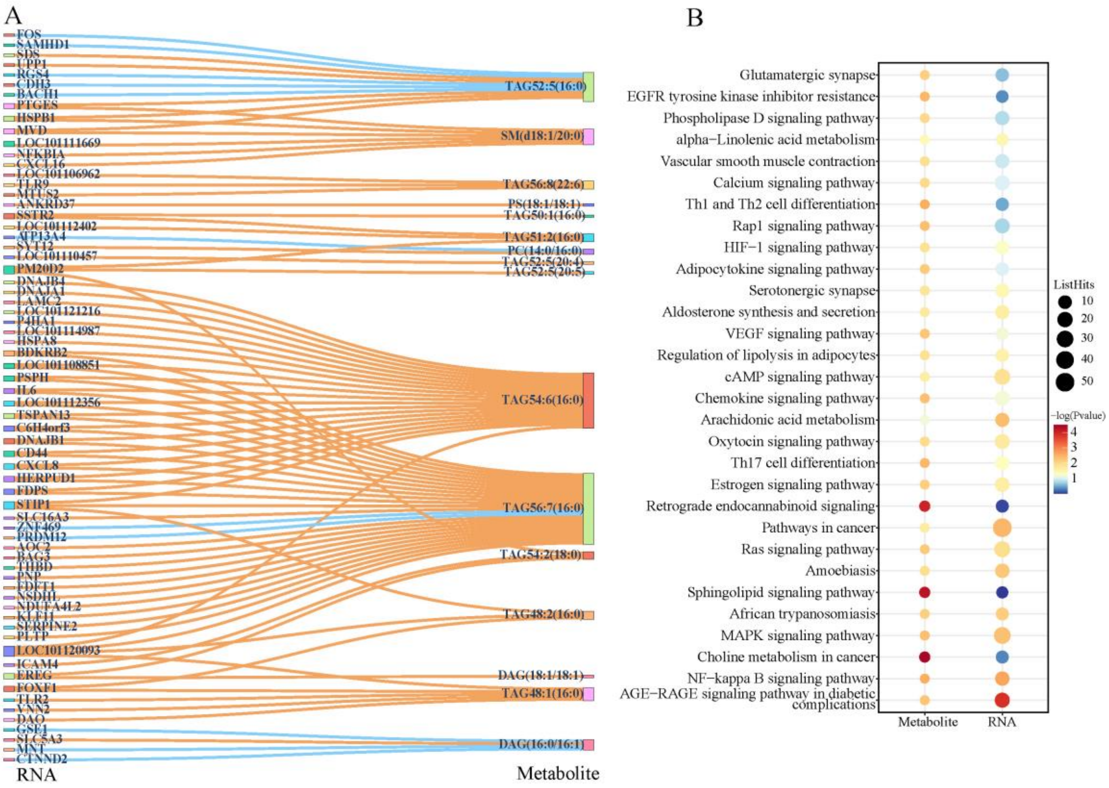

Figure S3 Joint analysis of transcriptome and metabolome. (A) Sankey diagram of the relationship between genes and metabolites. (B) Bubble diagram of the common mapping path of differentials.

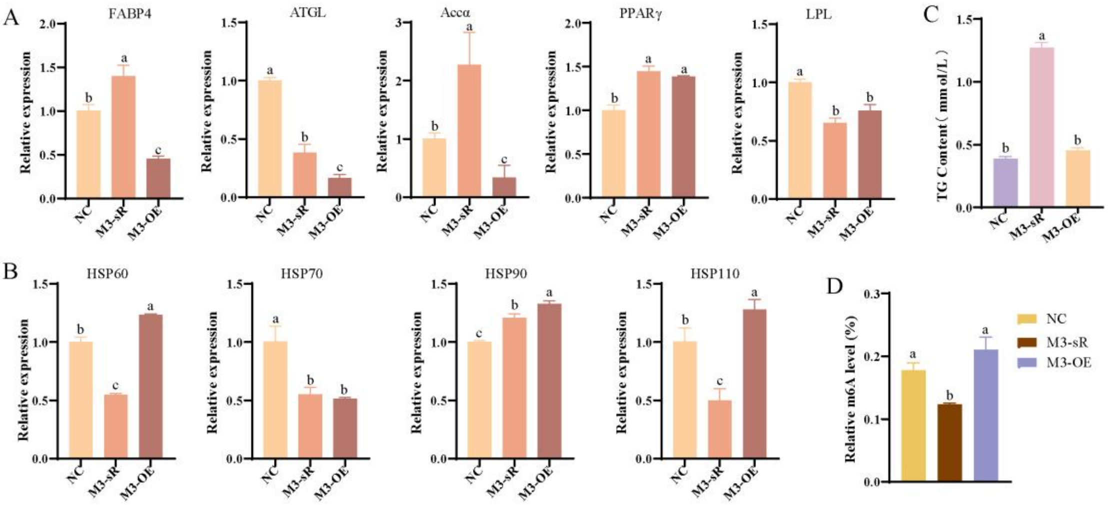

Figure S4 Effects of interference and overexpression of *METTL3* on ADHS of in preadipocytes. (A) Detection of lipid synthesis-related gene expression levels; (B) Detection of expression levels of heat stress-related genes; (C) Detection of m6A methylation level; (D) Detection of TG content.

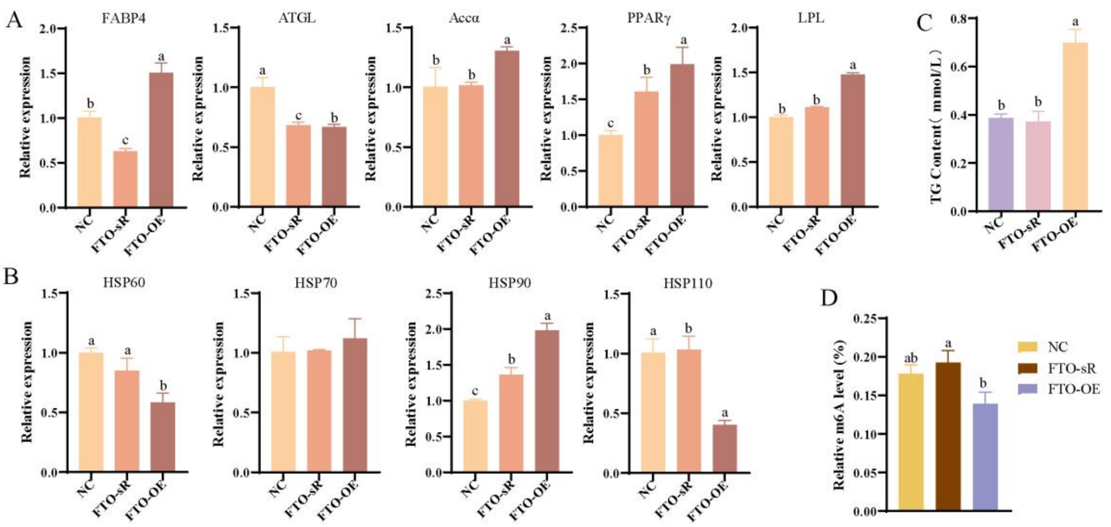

Figure S5 Effects of interference and overexpression of *FTO* on ADHS of in preadipocytes. (A) Detection of lipid synthesis-related gene expression levels; (B) Detection of expression levels of heat stress-related genes; (C) Detection of m6A methylation level; (D) Detection of TG content.
