# Supplementary material for: METTL3 and FTO Regulate Heat Stress Response in Hu Sheep Through Lipid Metabolism via m6A Modification
Source: Animals (Basel). 2025 Jan 13;15(2):193. doi: 10.3390/ani15020193 (PMC11758659; doi:10.3390/ani15020193)
Supplement: Supplementary file 1 [file animals-15-00193-s001.zip › Supplemental tables-revied.pdf]

Table S1 Primer sequence information

Gene	Primer sequences (5'-3')	Accession No.	Products length (bp)
METTL3	F: AGATAGAGAGCCTTCTTAACCA<br>R: TCACTGGCTTTCATACACTCC	XM_004010362.3	196
METTL14	F: ATATTACAGAGAGACCGGCAT<br>R: CAATCCTTCCCCAGAACCACA	XM_004009592.3	133
FTO	F: CATGGCTTCCCTACCTGACC<br>R: CTCGAAATAAACAGCCATGCTT	EU072419.1	165
YTHDF2	F: CCTGTAATTCTACTCGTGGTGA<br>R: AAATATTCTGCTGCACGTTG	XM_015097785.1	200
HSP60	F: CCTTAATGCTACACGAGCTG<br>R: AACACCTGCATTCTTAGCAA	XM_004004792.3	184
HSP90	F: CAGTTCATTGGCTATCCCAT<br>R: TCTTCATCTGAGCCAACGTCT	EF091713.1	164
HSP110	F: ACAAGCTGGAAGAACTAACGAA<br>R: AGTCAGCTGCTATCTTGGCAT	XM_015098145.1	129
PPARγ	F: ATAAAGCGTCAGGGTTCCAC<br>R: ATCCGACAGTTAAGATCACACC	NM_001100921.1	115
FABP4	F: CATAAACTTAGATGAAGGTGCTC<br>R: CACCGTTCATGACACATTCCA	EU301804.1	116
ATGL	F: CCGCTGCACCCTTCCTTCAACA<br>R: ACATTGGCCTGGATAAGCTCCT	KC768883.1	182
Accα	F: CCAGCAGAATTTGTTACTCGT<br>R: TAATGTATTCTGCGTTGGCTT	NM_001009256.1	196
LPL	F: AGACTCGTTCTCAGATGCCTT<br>R: CTCTCAGCCACAGTGCCAT	NM_001009394.1	127
ACTB	F: CTTCCAGCCTTCCTTCCTGG<br>R: GCCAGGGCAGTGATCTCTTT	NM_001009784.1	180

Table S2 Summary of sequencing RNA-seq data

Sample	RawReads (M)	CleanReads (M)	Q30 (%)	GC (%)
No_LDHS1	48.08	47.36	94.33	56.09
No_LDHS2	47.32	46.59	94.20	56.51
No_LDHS3	48.16	47.41	94.12	56.25
LDHS1	47.27	46.57	94.56	55.88
LDHS2	47.31	46.68	94.31	55.38
LDHS3	47.84	47.07	94.33	55.79
LV3_NC1	47.67	47.00	94.34	56.18
LV3_NC2	48.17	47.37	94.01	56.78
LV3_NC3	48.09	47.40	94.15	56.18
M3-sR-1	46.91	46.29	93.98	56.49
M3-sR-2	47.65	47.02	94.46	56.11
M3-sR-3	47.14	46.45	93.79	55.93
M14-sR-1	47.09	46.40	94.36	56.03
M14-sR-2	47.85	47.19	94.40	56.38
M14-sR-3	48.19	47.50	94.31	56.06
FTO-sR-1	47.29	46.56	94.08	56.27
FTO-sR-2	47.23	46.56	94.57	56.50
FTO-sR-3	47.40	46.78	94.21	56.17
LV5_NC1	47.26	46.64	94.13	56.06
LV5_NC2	47.07	46.40	94.30	56.51
LV5_NC3	47.26	46.61	94.42	56.35
M3-OE-1	47.70	47.00	93.88	56.38
M3-OE-2	48.18	47.41	94.34	56.00
M3-OE-3	47.81	47.17	93.80	55.90
